# Two-Step Surgical Strategy for Parathyroid Carcinoma: A Single-Center Experience

**DOI:** 10.3390/medicina60122054

**Published:** 2024-12-13

**Authors:** Filippo Longo, Antonio Sarubbi, Claudia Palladino, Andrea Palermo, Anda Mihaela Naciu, Anna Crescenzi, Chiara Taffon, Gaia Tabacco, Luca Frasca, Pierfilippo Crucitti

**Affiliations:** 1 Thoracic Surgery Department, Fondazione Policlinico Universitario Campus Bio-Medico, 00128 Rome, Italy; claudia.palladino@unicampus.it (C.P.); l.frasca@policlinicocampus.it (L.F.); p.crucitti@policlinicocampus.it (P.C.); 2 Master’s Degree Program in Medicine and Surgery, Campus Bio-Medico University of Rome, 00128 Rome, Italy; 3 Unit of Metabolic Bone and Thyroid Diseases, Fondazione Policlinico Universitario Campus Bio-Medico, 00128 Rome, Italy; a.palermo@policlinicocampus.it (A.P.); a.naciu@policlinicocampus.it (A.M.N.); g.tabacco@policlinicocampus.it (G.T.); 4 Unit of Endocrinology and Diabetes, Campus Bio-Medico University of Rome, 00128 Rome, Italy; 5 Unit of Endocrine Organs and Neuromuscular Pathology, Fondazione Policlinico Universitario Campus Bio-Medico, 00128 Rome, Italy; anna.crescenzi@uniroma1.it; 6 Department of Radiological, Oncological and Pathological Sciences, Sapienza University of Rome, 00161 Rome, Italy; 7 Pathology Unit, Fondazione Policlinico Universitario Campus Bio-Medico, 00128 Rome, Italy; c.taffon@policlinicocampus.it

**Keywords:** parathyroid carcinoma, primary hyperparathyroidism, hypercalcemia, parathyroidectomy, en bloc resection

## Abstract

Parathyroid carcinoma (PC) is a rare endocrine malignancy that poses significant diagnostic challenges due to its resemblance to benign conditions. This case series describes the clinical presentation, diagnosis, management, and short-term outcomes of four male patients (aged 54, 65, 73, and 74 years) with primary hyperparathyroidism and hypercalcemia. The preoperative diagnosis of PC remains challenging; suspicion should arise in cases of severe hypercalcemia, elevated parathyroid hormone levels, and the presence of a mass on imaging or during surgery. All patients underwent an initial localized parathyroidectomy, with PC confirmed postoperatively. Subsequently, they received ipsilateral hemithyroidectomy and prophylactic central lymph node dissection. Over a two-year follow-up period, all patients maintained normocalcemia without evidence of disease recurrence or metastasis. In conclusion, whether to perform a complete en bloc resection or a two-step surgical strategy remains a difficult decision in PC patients with intricate preoperative evaluations.

## 1. Introduction

Among rare malignancies, parathyroid carcinoma (PC) represents less than 0.1% of all types of cancers and less than 1% of all causes of primary hyperparathyroidism (PHPT) [1,2]. The etiology of PC is unclear, although it may be sporadic or part of a familial syndrome, such as hyperparathyroidism-jaw tumor (HPT-JT) and familial isolated primary hyperparathyroidism (FIPH). Loss-of-function of the Cell Division Cycle 73 (CDC73) tumor suppressor gene, encoding parafibromin, is the major driver of genetic defect [3].

The preoperative diagnosis of PC is particularly challenging, primarily because its clinical presentation closely resembles that of benign parathyroid adenomas (BPAs) [4,5]. PC should be suspected in patients with marked hypercalcemia (>14 mg/dL) and parathyroid hormone (PTH) levels 10 times or more than the upper limit [4]. Furthermore, patients with PC often exhibit more pronounced symptoms compared to individuals with parathyroid adenoma (PA), such as skeletal and renal complications [6]. The preoperative diagnosis of PC should also consider the evidence of lymph node metastasis, invasive tumor growth at histology, and/or distant metastases. However, histopathologic confirmation after surgery is the only method to confirm the diagnosis [7].

The en bloc resection of the tumor, ipsilateral thyroid lobe, and adjacent structures is the gold standard, reducing recurrence risk and enhancing long-term outcomes [2]. Nevertheless, PC carries a recurrence rate of 40% to 60%, and multiple surgical interventions are often needed [5]. Other treatment options such as radiation therapy, chemotherapy, or immunotherapy are limited [8,9]

This study aims to describe the clinical features, diagnostic approach, and management of PC, assessing the efficacy of a two-step surgical strategy in achieving optimal disease control and minimizing recurrence.

## 2. Materials and Methods

Four male patients with asymptomatic PHPT were included in this series. We obtained written informed consent from all patients. Combined imaging with neck ultrasound (US) and 99mTc-Sestamibi (MIBI) were performed in all patients. All surgeries were conducted by the same team to ensure consistency in surgical approach and outcomes. The patients’ characteristics, radiological findings, and surgical management are listed in Table 1. Moreover, we conducted a focused review of the recent literature, examining similar case reports or studies on the surgical management of PC.

## 3. Results

The mean age was 66.5 ± 9.25 years (range 54–74). None had any family history of hyperparathyroidism or cancer. A history of nephrolithiasis was present in two cases, while the other subjects were totally asymptomatic.

At first inspection, all subjects were characterized by palpable neck masses, hard in consistency. Laboratory findings indicated preoperative serum calcium and PTH levels of 13.3 ± 1.85 mg/dL (range 11.6 to 15 mg/dL) and 620.95 ± 528.3 pg/mL (range 126 to 1367.3 pg/mL), respectively. The remaining blood tests were normal, and the serum thyroid function panel was within limits.

Both preoperative US and MIBI scans were performed on all the patients. Neck US showed a regularly shaped hypoechoic nodule, with solid components and multiple blood flow signals located at the ipsilateral lower pole of the thyroid. MIBI scans consistently exhibited a metabolic tracer uptake corresponding to these lesions. Overall, this combined imaging technique correctly localized the site of the tumor. Furthermore, fine-needle aspiration biopsy (FNAB) with a PTH assay on needle lavage fluid was performed in one case to differentiate between the suspected functional PA and a thyroid nodule. The resulting PTH levels (>2000 pg/mL) confirmed the parathyroid origin of this lesion.

In all patients, a hard mass was noted intraoperatively, and a simple parathyroidectomy was performed. In each patient, we routinely observed a firm decrease in PTH levels at 20’ after excision, indicating a complete lesion excision.

Surgical procedures concluded without complications and all the patients were discharged on the third postoperative day.

**Figure 1:**
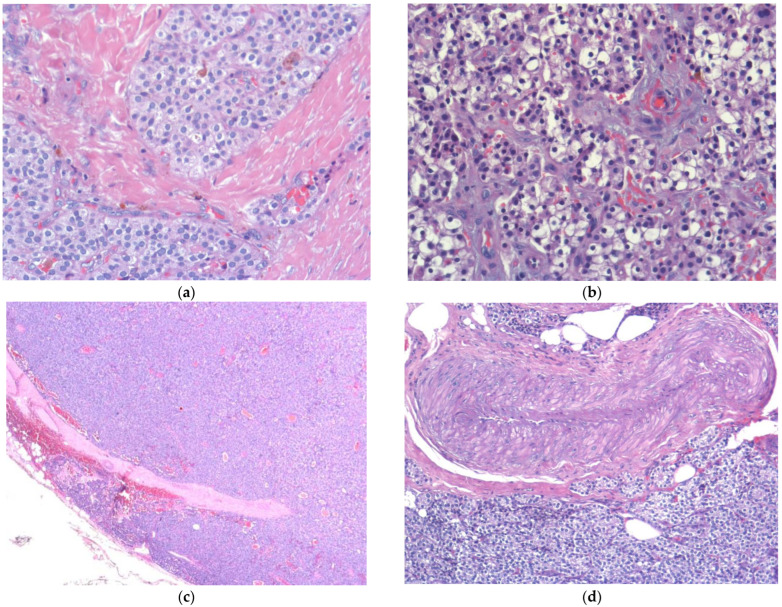
Parathyroid carcinoma: histopathological findings. (**a**) Solid tumor with sclerosis and cellular atypia observed under 20× field; (**b**) clear cells (EE 20×); (**c**) extracapsular invasion (EE 2×); (**d**) perivascular invasion (EE 10×).

In all cases, the diagnosis of PC was established postoperatively following histopathological examination (Figure 1). The latter revealed chief cell PC in three cases and an oxyphil cell carcinoma displaying a solid growth pattern in one case. In those patients, the neoplasm infiltrated the fibrous capsule and was focally present at the specimen margins (R1): in all of them, a re-exploration with ipsilateral hemithyroidectomy was performed along with prophylactic CLND. The isolated lymph nodes were negative, thyroid parenchyma was free from disease, and resection margins were now clear.

**Figure 2:**
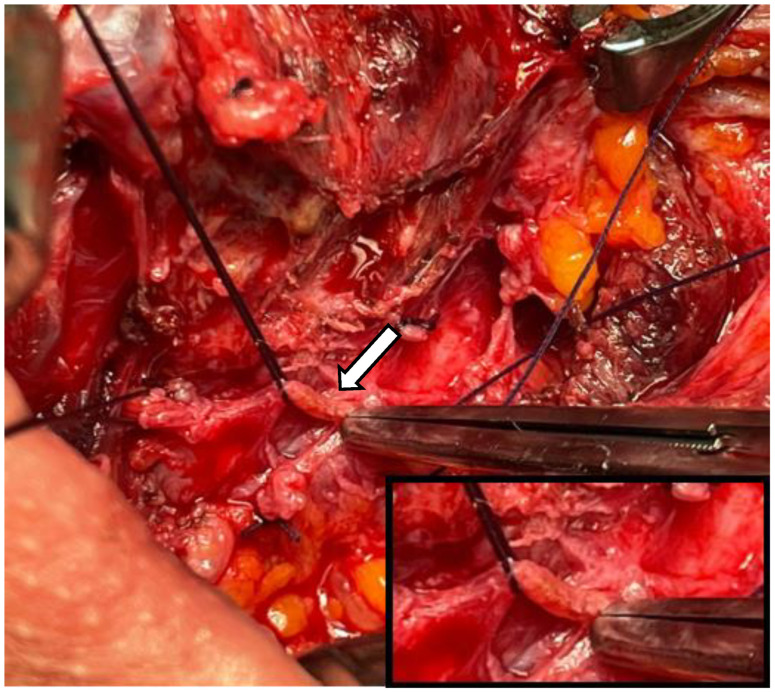
Patient 3: Intraoperative view of recurrent laryngeal nerve. The arrow points to a dyschromic area in which biopsy was performed soon after parathyroidectomy, showing infiltration by a carcinoma.

In one case, signs of prior neoplastic involvement were identified in the esophageal branch of the right recurrent laryngeal nerve (RLN) and adjacent tissues (Figure 2). Consequently, the patient underwent re-exploration with radical intent: neurotomy was performed, followed by termino-terminal fiber anastomosis. At discharge, the patient experienced right-sided laryngeal paralysis, which was effectively managed with corticosteroids. All patients were normocalcemic after surgery. All patients were followed up every 6 months for 2 years. At 1 month postoperatively, calcium and PTH levels were 9.42 ± 0.23 mg/dL (range 9.1–9.6 mg/dL) and 66 ± 12.49 pg/mL (range 53–81 pg/mL), respectively. The 2-year follow-up included US assessment and the regular monitoring of calcium/PTH levels and symptoms. At present, all patients are alive and have not shown any signs of residual or recurrent disease.

## 4. Discussion

PC is a rare slow-growing tumor responsible for 0.1% to 5% of cases of PHPT [1,10]. Approximately 90% of PCs are functional, meaning that they produce excessive PTH which leads to hypercalcemia. The differential diagnosis between PC-induced PHPT and PHPT due to benign diseases is often challenging [11].

PC should be suspected whenever patients with a history of PHPT display one or more characteristics: (1) Palpable neck mass. We included in our study four patients with PHPT, characterized by neck swelling at physical observation. BPAs are essentially non-palpable, except for huge tumors [6]. (2) Symptomatic hypercalcemic symptoms, such as asthenia, depression, gastrointestinal disorders, nephrolithiasis, pain, or bone fractures [6]. One patient presented with nephrolithiasis, whereas the others were totally asymptomatic. Some patients may present with a hypercalcemic crisis (>16 mg/dL), with severe renal and skeletal manifestations frequently reported. (3) Serum calcium values greater than 14 mg/dL and PTH levels 5–15 times higher than normal [4]. In our experience, severe hypercalcemia with a PTH value of more than 10–15 times the upper limit coexisted in two patients, whereas the other two were not biochemically suggestive of carcinoma (calcium <14 mg/dL), even though their PTH levels were extremely high. Therefore, laboratory findings are not specific, and a standard threshold of malignancy is still under debate [2]. (4) Lobulated, hypoechoic, and relatively larger nodules with irregularly defined borders at US [2]. Biochemical profiles must be integrated with imaging studies [4]. US is the gold standard approach to localize primary and locally recurrent disease [12]. Combined imaging with neck US and MIBI scans has a 100% sensitivity for PC localization [13]. In our series, both exams were performed: in all cases, they correctly localized the site of the neoplasm prior to surgery. All patients had suspicious US findings, including lobulated, hypoechoic, and relatively larger nodules with irregular defined borders. Computed tomography (CT), magnetic resonance imaging (MRI) and positron emission tomography (PET) may be useful in the identification of metastases and recurrence [13]. Moreover, FNAB is not recommended due to its potential to increase the risk of recurrence following tumor rupture and seeding [14]. However, in one case, we performed FNAB with a PTH assay on the needle lavage fluid (PTH: 200 pg/mL). This procedure provided an accurate assessment of the hormonal activity within the suspected lesion.

Surgical resection is the optimal treatment for PC; however, there is no consensus on the appropriate extent of surgery [15]. If there is a strong suspicion of PC before surgery, most of the authors recommend proceeding with the en bloc resection of the tumor, including the adjacent thyroid and any infiltrated tissues [9].

**Figure 3:**
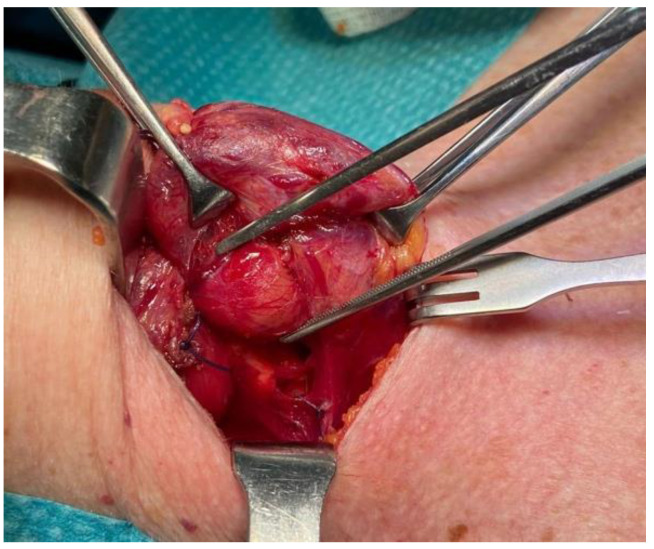
Intraoperative view during surgery of a solid, firm, large mass; in the mid-third region of the right thyroid lobe, hyperplastic parathyroid tissue measuring approximately 15 mm in diameter was observed. Following histological analysis revealed the benign nature of the neoplasm.

Intraoperatively, if the gland is large, firm, white, or hard, and if there is local invasion, the diagnosis of cancer is suggestive, but not definitive [5,6,14]. In our experience, these macroscopical features guided us to perform an en bloc resection of the neoplasm, along with ipsilateral hemithyroidectomy and CLND, in a patient (not included in the present series) with hypercalcemic crisis (serum calcium: 16.9 mg/dL). Despite the strong suspicion of PC, subsequent final histology revealed the benign nature of the neoplasm (Figure 3). 

On the other hand, other studies support a less extensive approach, which involves performing a localized parathyroidectomy to remove the suspected lesion, in cases where disease localization suggests limited spread and less aggressive behavior [14,15,16].

A common scenario involves identifying PC postoperatively in a patient initially suspected to have a benign condition [9]. In the present series, four patients were diagnosed with PC after the removal of the gland. In these patients, we performed a localized ipsilateral parathyroidectomy, since there were no intraoperative signs of adhesions or invasion of the surrounding tissue, including the thyroid. The histopathologic results of all patients corresponded with the criteria for PC.

Recurrences typically occur within 2 to 3 years after the initial surgery and are reflected by elevated PTH and calcium levels identified at follow-up [9], although timely reoperation within one month reduces recurrence and offers a second chance for a cure [17]. Therefore, this approach inevitably necessitates a neck re-entry with ipsilateral hemithyroidectomy and prophylactic CLND (two-step surgical strategy) to achieve radicality. It has not yet been established that preventive CLND is effective in increasing survival [9].

While nodal involvement (15–20%) and distant metastasis (1%) are potential concerns, some studies suggested that revision surgery may not always be immediately necessary, advocating for close monitoring as a viable alternative [5,15,18]. Upon reoperation, we found thyroid tissue free from neoplastic involvement, raising doubts about the necessity of immediate revision surgery in those cases.

Extreme caution should be taken to avoid disrupting the capsule, since the seeding of the tumor has been widely reported [19]. Moreover, obtaining clear margins is fundamental to achieve disease control and eliminate residual disease [9,15]. In one patient, for instance, revision surgery increased morbidity by cutting the RLN fibers due to prior neoplastic involvement.

Although there is significant debate as to which surgical technique should be performed, a systematic review led by P. Roser et al. concluded that primary en bloc resection is the gold standard in those cases in which PC is suspected [2]. This technique is favored over simple parathyroidectomy as it offers the complete excision of the tumor with clear margins, which decreases the likelihood of locoregional recurrence [16].

Nevertheless, localized surgery may preserve thyroid function and minimize the risk of iatrogenic complications and failure associated with a second neck exploration [14]. These include a higher incidence of RLN palsy, hypoparathyroidism, increased scar tissue, and wound complications. However, reoperation is more favorable than no intervention, and patients should be referred to surgeons experienced in thyroid and parathyroid surgery [20].

Finally, this study has several limitations that deserve consideration. First, the small sample size and single-center, retrospective design may limit the generalizability of our findings. Additionally, the absence of a control group and the relatively short follow-up duration could affect the robustness and applicability of our conclusions.

## 5. Conclusions

Parathyroid carcinoma (PC) is a rare malignancy. Our limited experience confirms that diagnosing and treating PC is challenging due to its rarity and diagnostic overlap with benign conditions. While complete resection is the gold standard in all patients with suspected PC, debate exists over the necessity of further revision surgery in patients diagnosed with PC following parathyroidectomy (two-step surgical strategy). Incomplete excision may lead to an increased risk of recurrence, thus emphasizing the importance of close follow-up in the context of PC. Further multicenter, prospective studies are needed to validate these results and to improve the knowledge and treatment of this cancer.

## Figures and Tables

**Table 1:** Overview of clinical, radiological, and biochemical findings, treatment, and outcomes of our patients.

	Patient 1	Patient 2	Patient 3	Patient 4
Age (years), Gender	73 M	54 M	65 M	74 M
Presentation	Palpable neck mass	Palpable neck mass, nephrolithiasis	Palpable neck mass	Palpable neck mass
Serum calcium	15 mg/dL	11.6 mg/dL	11.8 mg/dL	14.8 mg/dL
Intact PTH (iPTH)	1367.3 pg/mL	126.0 pg/mL	452.0 pg/mL	538.5 pg/mL
US diameters (mm)	13 × 15 × 14	13 × 23 × 25	15 × 16 × 20	22 × 17 × 14
MIBI scan	+	+	+	+
Thyroid	Normal	Normal	Normal	Normal
Operation	R inferior PTX	R inferior PTX	R inferior PTX	L inferior PTX
Histology	PC	PC	PC	PC
Invasion	Capsule	Capsule	Capsule, RLN	Capsule
Ki-67 proliferation index (%)	6	12	4	4
R1	Yes	Yes	Yes	Yes
Revision surgery ^a^	Yes	Yes	Yes	Yes
Lymph node metastasis	N0	N0	N0	N0
Complications	No	No	RLN neurotomy, RLN palsy	No
Thyroid parenchyma	NED	NED	NED	NED
Final diagnosis	pT1, pN0 pM	pT1, pN0 pM	pT1, pN0 pM	pT1, pN0 pM
IO PTH dropping at 20’ (%)	80	48	96	
POM calcium results	9.4 mg/dL	9.6 mg/dL	9.1 mg/dL	9.6 mg/dL
POM PTH results	81 pg/mL	71 pg/mL	59 pg/mL	53 pg/mL
2y FU (PTH- + Ca-Levels + US)	NED	NED	NED	NED

Reference range serum total calcium: 8.4 mg/dL to 10.2 mg/dL; reference range PTH: 13 pg/mL to 85 pg/mL. IO: intraoperative; PTX: parathyroidectomy; RLN: recurrent laryngeal nerve; POM: postoperative month; NED: no evidence of disease. ^a^ Revision surgery included hemithyroidectomy and central lymph node dissection on the side of the parathyroid cancer.

## Data Availability

The datasets generated and/or analyzed during the current study are available from the corresponding author on reasonable request.
